# Single Cell Self-Paced Clustering with Transcriptome Sequencing Data

**DOI:** 10.3390/ijms23073900

**Published:** 2022-03-31

**Authors:** Peng Zhao, Zenglin Xu, Junjie Chen, Yazhou Ren, Irwin King

**Affiliations:** 1 School of Computer Science and Engineering, University of Electronic Science and Technology of China, Chengdu 611731, China; penn201@std.uestc.edu.cn; 2 School of Computer Science and Technology, Harbin Institute of Technology Shenzhen, Shenzhen 518055, China; junjiechen@hit.edu.cn; 3 Center of Artificial Intelligence, Peng Cheng National Lab., Shenzhen 518066, China; 4 Institute of Electronic and Information Engineering of UESTC in Guangdong, Dongguan 523808, China; 5 Department of Computer Science and Engineering, Chinese University of Hong Kong, Hong Kong 999077, China; king@cse.cuhk.edu.hk

**Keywords:** sequencing data, scRNA-seq, clustering, self-paced learning, nonnegative matrix factorization

## Abstract

Single cell RNA sequencing (scRNA-seq) allows researchers to explore tissue heterogeneity, distinguish unusual cell identities, and find novel cellular subtypes by providing transcriptome profiling for individual cells. Clustering analysis is usually used to predict cell class assignments and infer cell identities. However, the performance of existing single-cell clustering methods is extremely sensitive to the presence of noise data and outliers. Existing clustering algorithms can easily fall into local optimal solutions. There is still no consensus on the best performing method. To address this issue, we introduce a single cell self-paced clustering (scSPaC) method with F-norm based nonnegative matrix factorization (NMF) for scRNA-seq data and a sparse single cell self-paced clustering (sscSPaC) method with $l_{21}$-norm based nonnegative matrix factorization for scRNA-seq data. We gradually add single cells from simple to complex to our model until all cells are selected. In this way, the influences of noisy data and outliers can be significantly reduced. The proposed method achieved the best performance on both simulation data and real scRNA-seq data. A case study about human clara cells and ependymal cells scRNA-seq data clustering shows that scSPaC is more advantageous near the clustering dividing line.

## 1. Introduction

Single cell RNA sequencing (scRNA-seq) is a powerful new approach for studying the transcriptomes of cell lines, tissues, tumors and disease states. The use of scRNA-seq has already yielded key biological insights and discoveries, such as a better knowledge of cancer tumor heterogeneity [1]. In recent years, advances in scRNA-seq have promoted the study of computational methods for analyzing transcriptome data from single cells. Since the information about sequential cells is only partial, cluster analysis is usually used to discover cell subtypes or to distinguish and better characterize known cell subtypes [2]. However, the analysis methods are typically complex, and the user is often simply given a visual representation of the data with no assessment of the robustness of the groupings.

Unlike bulk RNA-seq data, single cell RNA-seq data are more sparse and have a high dropout rate, which makes clustering very challenging. Recently, several methods and tools have been developed for single cell RNA-seq clustering. K-means is used in several approaches for evaluating scRNA-seq data. In rounds of grouping single cells, single cell analysis via iterative clustering (SAIC) [3] combines K-means and analysis of variance, followed by signature gene identification. Single cell clustering using bifurcation analysis (SCUBA) [4] divides cells into two groups at each time point using K-means, and then utilizes gap statistics to locate bifurcation occurrences. The method in [5] uses non-negative matrix factorization to incorporate information from a larger annotated dataset and then applies transfer learning to perform the clustering. Clustering through imputation and dimensionality reduction (CIDR) uses hierarchical clustering to do data imputation before clustering a principal component analysis (PCA)-reduced representation [6]. Semisoft clustering with pure cells (SOUP) can handle both pure and transitional cells and computes soft cluster memberships using the expression similarity matrix [7]. Maaten et al. [8] introduced a novel embedding algorithm named the t-distributed stochastic neighbor embedding (t-SNE) algorithm. The t-SNE is a dimensionality reduction method that may also be used to classify single cells. The spectral clustering (SC) algorithm finds a low-dimensional embedding of data by calculating the eigenvectors of the constructed Laplacian matrix [9] and is one of the most widely used algorithms for data clustering. Hu et al. [10] proposed a new low-rank matrix factorization model for scRNA-seq data clustering based on sparse optimization. Wang et al. [11] developed a novel single cell interpretation via multi-kernel learning (SIMLR) method to construct the similarity matrix by fusing multiple Gaussian kernel functions, and it clusters the single cells by applying the spectral clustering algorithm to the similarity matrix. To characterize the sparsity of scRNA-seq data, Part et al. [12] improved the SIMLR method by integrating doubly stochastic affinity matrices and sparse structure constraints to cluster single cells.

Self-paced learning (SPL) [13] is a novel machine learning framework that has recently gained a lot of interest. The concept is based on the principle that individuals learn better when they begin with simple knowledge and work their way up to more complicated knowledge. Bengio et al. presented curriculum learning to define this method in machine learning (CL) [14]. After that, Kumar et al. [13] suggested using SPL for curriculum design purposes by including an SPL regularization term in the objective function. The learning difficulty of the instances (either simple or complex) depends on the loss of the current parameter values. The capacity of SPL to avoid undesirable local minima and so have superior generalization ability has been empirically shown [13,15,16,17,18]. The authors of [19] used SPL to solve non-convex problems caused by feature destruction techniques. Traditional clustering algorithms are either easily caught in local optima or susceptible to outliers and noisy data [20,21,22,23]. Ren et al. [22] proposed a unique self-paced multi-task clustering (SPMTC) method to address these issues in multi-task clustering. Yu et al. [23] offered a self-paced, learning-based K-means clustering method. To deal with the non-convex problem in multi-view clustering, DSMVC [24] uses self-paced learning. Therefore, SPL is often used to find better solutions for non-convex problems.

**Figure 1:**
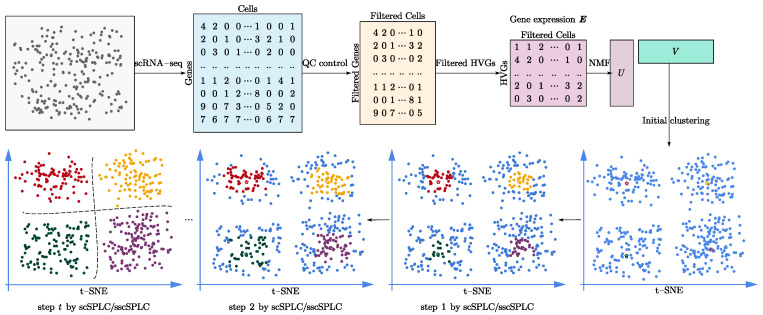
Workflow for single cell self-paced clustering (scSPaC) and sparse single cell self-paced clustering (sscSPaC), which included data preprocessing, clustering and visualization. The pentagram in the figure represents the cluster center. The number of clusters is searched within a reasonable range (determined by an existing tool, SCANPY), and we discuss the impact of the cluster number on model performance in Section 3.3.

Due to the non-convexity of nonnegative matrix factorization (NMF) models for scRNA-seq clustering, these models easily obtain a bad local solution. In this study, we introduce a single cell self-paced clustering (scSPaC) model and a sparse ($l_{21}$-norm based) single cell self-paced clustering (sscSPaC) model. Specifically, single cells are gradually incorporated into the NMF process from simple to complex, which draws on the advantages of SPL and has been shown to help models avoid falling into local minima. In our other model, i.e., sscSPaC, $l_{2,1}$-norm is used, which reduces the effects of noise and outliers. In order to verify the effectiveness of the introduced methods, we conducted comparative experiments on simulation data and real scRNA-seq data. The workflow of this study is shown in Figure 1, including data preprocessing, clustering and visualization.

## 2. Materials and Methods

### 2.1. Datasets

To illustrate the efficacy of the two novel scRNA-seq clustering algorithms in further detail, on simulated and real single cell data, we compared the performances of these two clustering algorithms and baselines. We generated simulated data to evaluate the clustering performance of scSPaC. Splatter [25], a tool commonly used to generate scRNA-seq data, was utilized to generate the experimental data. Simulation data were obtained from two classes with 100 single cells per class. Each cell contains 22,002 genes. The real datasets are described in the following: baron [26], kolodziejczyk [27], pollen [28], rca [29], goolam [30], zeisel [31], and cell lines [32], which includes a mixture of 1047 cultured human BJ, H1, K562 and GM12878 cells. The statistical information of all datasets used in this study is shown in Table 1. The datasets contain 2–14 cell types, and the number of cells in each dataset ranges from 124 to 3500. The number of genes in each of these datasets exceeds 10,000. The maximum is 32,316 genes.

### 2.2. Data Preprocessing

Raw scRAN-seq read count data are sparse and high-dimensional, which makes further subsequent statistical analysis challenging [33]. Therefore, we needed to pre-process the raw matrix data. The raw data were pre-processed by the Python package Scanpy [34] as follows:

Step 1: Genes with no count in any cell were filtered out.
Step 2: We filtered genes that were not expressed in almost all cells.
Step 3: The top N high variable genes (HVGs) were selected. One thousand highly variable genes were selected by default. In Section 3.2. We discuss the influence of different N values for the experimental accuracy.
Step 4: The last step was to take the log transform and scale of the read counts, so that count values follow unit variance and zero mean.

The pre-processed read count matrix was treated as the input for our scSPaC model and the other algorithms.

### 2.3. scSPaC Model

Consider a log-transformed count matrix $X=\left[x_{1},x_{2},\cdots ,x_{n}\right]\in R^{m\times n}$, where *n* is the number of cells and *m* is the number of genes. Nonnegative matrix factorization (NMF) [35] aims to find two nonnegative matrices $U\in R^{m\times r}$ and $V\in R^{r\times n}$, which minimizes the following objective function:

(1) $$\begin{array}{c} J_{1}={∥X-{\mathrm{UV}}^{T}∥}_{p}\\ s.t.\;U\geq 0,V\geq 0, \end{array}$$

where ${∥\cdot ∥}_{p}$ is p-norm. $X_{ij}$ in X denotes the gene expression of the *i*-th gene in the *j*-th cell. V can be regarded as the new representation of the original data with respect to the new basis U. *r* represents the components of U and V. Lee et al. [35] proposed an algorithm for iteratively updating U and V to optimize the objective (Equation (1)). It adopts the Frobenius norm (F-norm) NMF model, which is sensitive to noisy data [36,37]. Recently, the authors of [36] proposed robust NMF methods with $l_{2,1}$-norm. Compared with the F-norm NMF, the $l_{2,1}$-norm NMF is robust to noisy data, since the non-squared residuals $\{∥x_{i}-Uv_{i}{∥}_{2}{\}|}_{i=1}^{n}$ reduce the effects of outliers [36].

To mitigate the tendency of NMF model to fall into a local optimum solution, we introduce a SPL regularization term to NMF model for scRNA-seq clustering.

(2) $$\begin{array}{c} \min_{U,V,w}{∥diag\left(w\right)\left(X-{\mathrm{UV}}^{T}\right)∥}_{p}+f\left(\lambda ,w\right)\\ s.t.\;U\geq 0,V\geq 0,w\in {\left[0,1\right]}^{n}, \end{array}$$

where diag(w) denotes a diagonal matrix with the *i*-th diagonal element being $w_{i}$. One of the simple regular functions f(λ,w) is shown in Equation (3). Kumar et al. [13] proposed to let w∈{0,1} and define f(λ,w) as

(3) $$f\left(\lambda ,w\right)=-\lambda \sum_{i=1}^{n}w_{i},$$

Then, the optimal $w^{*}$ can be calculated by

(4) $$w_{i}^{*}=\left\{\begin{array}{cc} 1, & \mathrm{if}\;l_{i}<\lambda \\ 0, & \mathrm{otherwise} \end{array}.\right.$$

Since $w_{i}\;\left(i=1,\ldots ,n\right)$ is either 1 or 0, the strategy mentioned above can be treated as hard weighting. λ>0 is initially tuned to a small value such that the single cells with small loss values can be selected to clustering model. With the increasing of λ, more and more cells will be selected until all cells are chosen.

In Equation (2), if the p-norm is specific to the F-norm, we name the single cell clustering model scSPaC. This strategy has been successfully applied in the field of face recognition [38]. If the p-norm is specific to the sparse $l_{2,1}$-norm, the model is named sscSPaC.

The core idea of scSPaC and sscSPaC introduced in this work is to gradually select cells for decomposition from simple to complex.

Reference [39] proposed that Equation (2) with $l_{2,1}$-norm can be written as follows in simple algebra.

(5) $$\begin{array}{c} \min_{U,V,w}\mathrm{Tr}\left(\left(X-{\mathrm{UV}}^{T}\right)W{\left(X-{\mathrm{UV}}^{T}\right)}^{T}\right)+f\left(\lambda ,w\right)\\ s.t.\;U\geq 0,V\geq 0,V^{T}V=I,w\in {\left[0,1\right]}^{n}, \end{array}$$

where W is a diagonal matrix and $W_{ii}=w_{i}$.

### 2.4. Optimization

We utilize an iterative updating algorithm to solve the optimization problem of scSPaC and sscSPaC. Specifically, we iteratively optimize each variable in the objective function while fixing the other variables.

 **Step 1:** Fix w, update U and V.

When we fix w, f(λ,w) in Equation (2) is a constant. Solving Equation (2) is equivalent to solving the original NMF model Equation (1). Thus, we can update the model parameters U and V iteratively.

(a) Update U and V for the scSPaC model.

For Equation (1), Lee et al. [35] proposes an algorithm for iteratively updating U and V to optimize the objective.

(6) $$\begin{array}{c} U_{ij}^{\left(a\right)}\;\leftarrow \;U_{ij}\frac{{\left(XV\right)}_{ij}}{{\left(UV^{T}V\right)}_{ij}}, \end{array}$$

(7) $$\begin{array}{c} V_{ij}^{\left(a\right)}\;\leftarrow \;V_{ij}\frac{{\left(X^{T}U\right)}_{ij}}{{\left(VU^{T}U\right)}_{ij}}. \end{array}$$

(b) Update U and V for the sscSPaC model.

For Equation (5), we propose update rules for U and V as follows [39]:

(8) $$\begin{array}{c} U_{ij}^{\left(b\right)}\;\leftarrow \;U_{ij}\sqrt{\frac{{\left(XWV\right)}_{ij}}{{\left(UV^{T}WV\right)}_{ij}}}, \end{array}$$

(9) $$\begin{array}{c} V_{ij}^{\left(b\right)}\;\leftarrow \;V_{ij}\sqrt{\frac{{\left({\mathrm{WX}}^{T}U\right)}_{ij}}{{\left({\mathrm{WVV}}^{T}X^{T}U\right)}_{ij}}}. \end{array}$$

 **Step 2**: Fix U and V, update w.

With the fixed parameters U and V, the weight matrix diag(w) is updated by

(10) $$w^{*}=\arg\min\sum_{i=1}^{n}w_{i}l_{i}+f\left(\lambda ,w\right),$$

where the loss function $l_{i}={∥x_{i}-Uv_{i}∥}_{2}$ in Equation (2) is a constant. We can observe from Equation (3) that SPL chooses single cells based on their loss values and a parameter λ.

We consider assigning weights and gradually choosing single cells from simple to complex. For the single cell clustering problem, we define a new method for computing the hard and easy samples in self-paced learning. We define this single cell close to its own clustering center (i.e., far from other clustering centers) as a single cell that is easy to cluster and will be preferentially selected for the clustering model. We chose to utilize a new SPL regularization term.

The regularization term is defined as

(11) $$f\left(\lambda ,w\right)=-\sum_{i=1}^{n}\zeta \ln\left(w_{i}+\zeta /\lambda \right),$$

and the optimal $w^{*}$ is computed by

(12) $$w_{i}^{*}=\left\{\begin{array}{cc} 1, & \mathrm{if}\;l_{i}\leq \zeta \lambda /\left(\zeta +\lambda \right)\\ 0, & \mathrm{if}\;l_{i}\geq \lambda \\ \zeta /l_{i}-\zeta /\lambda , & \mathrm{otherwise} \end{array}.\right.$$

Equation (12) is a soft weighting strategy. According to [40], Equation (12) is also called mixture weighting. We set ζ=0.5×λ for simplicity in our experiments.

Now, we have all the update rules done. We optimize the model in an iterative way; i.e., steps 1 and 2 are iteratively repeated until the model convergence. We increase λ to select more single cells to the factorization process. Specifically, we initialize λ such that more than half (the default value is sixty percent) the cells are picked in the first iteration. In the following iteration, λ is increased such that 10% more cells can be added. As a consequence, λ is automatically determined. The model repeats until all the single cells are chosen. Finally, K-means clustering is applied to the matrix *V* after iteration, and the clustering results of scRNA-seq data are obtained. The clustering results will be evaluated and analyzed in the experimental section.

### 2.5. Evaluation Metrics

All clustering results are measured by adjusted rand index (ARI), purity and normalized mutual information (NMI). These cluster evaluation indicators will be briefly introduced here.

#### 2.5.1. ARI

Rand index (RI) [41] is a measure of similarity between two clusters. We can use it to compare actual class labels *C* and predicted cluster labels *Y* to evaluate the performance of a clustering algorithm. The adjusted rand index (ARI), described in formula (13), is the corrected-for-chance version of the rand index [42].

(13) ARI(C,Y)=∑ijnij2−[∑iai2∑jbj2]/N212[∑iai2+∑jbj2]−[∑iai2∑jbj2]/N2.

Here, *N* represents the number of all cells. $n_{ij}$ represents the number of cells that are in class *i* after clustering and should actually be in class *j*. $a_{i}$ denotes the logarithm of elements of the same cluster in both clusters *C* and true classes *Y*. $b_{j}$ denotes the logarithm of elements of different clusters in both clusters *C* and true classes *Y*. mk is standard *m*-choose-*k* notation. ARI ranges from −1 to 1. Perfect labeling is scored 1; bad clustering has negative or close to 0 scores. A larger value means that the clustering results match the real cell types better.

#### 2.5.2. Purity

Purity [43] is quite simple to calculate. It is applied to measure the extent to which each cluster contains data instances from primarily one class. The purity of a clustering result is computed by the weighted sum of each cluster purity values and can be defined as follows:

(14) $$Purity\left(C,Y\right)=\frac{1}{N}\sum_{k}\max_{j}\left|c_{k}⋂y_{j}\right|,$$

where $C=\left\{c_{1},c_{2},...c_{K}\right\}$ represent *K* different clusters, and $Y=\left\{y_{1},y_{2},...,y_{J}\right\}$ represent *J* different true classes. For Purity∈[0,1], the higher the value, the better the clustering result.

#### 2.5.3. NMI

Normalized mutual information (NMI) [44] measures the amount of information obtained about one partition through observing the other partition, ignoring the permutations:

(15) $$NMI\left(C,Y\right)=\frac{2I(C,Y)}{\left[H(C)+H(Y)\right]},$$

where H(.) is the entropy, and I(Y,C) measures the mutual information between *Y* and *C*.

## 3. Results and Discussion

### 3.1. Experimental Performance on All Datasets

The recently published benchmark article, Qi et al. [45], tested five representative clustering methods (SC3, SNN-Cliq, SINCERA, SEURAT, and pcaReduce) of the most advanced scRNA-seq tools and showed that SC3 had the highest clustering accuracy under default parameters. Seurat performed well in the mixture control experiment reported by the recently published benchmark article [46]. Scanpy is a widely used python package for single cell analysis [47]. Therefore, we only compared our scSPaC and sscSPaC with SC3, Scanpy and Seurat, three basic NMF models; and the K-means method. To ensure that comparisons between algorithms were based on the same criteria, we used the same gene-filtering and normalization steps for all these algorithms. The main steps of data preprocessing are shown in Section 2.2.

To evaluate the performances of the proposed scSPaC and sscSPaC, we compared them with several closely related nonnegative matrix factorization (NMF) methods and scRNA-seq clustering tools:

1. K-means [48], the classical K-means algorithm.
2. NMF [35], the standard NMF clustering with Frobenius norm (F-norm).
3. ONMF [49], the orthogonal NMF for clustering.
4. $l_{2,1}$-NMF [36], the sparse NMF clustering with $l_{2,1}$-norm.
5. Scanpy [34] is a Python-based toolkit for analyzing single cell gene expression data. Scanpy was downloaded from https://github.com/theislab/scanpy (accessed on 3 March 2022). It includes clustering and is used as the comparison algorithm in the experiment. We ran Scanpy with default parameters, for example, n_neighbors=20 and resolution=1.0.
6. Seurat3 [50] is a graph-based clustering tool. For all datasets, Seurat was performed with default parameters and downloaded from https://github.com/satijalab/seurat (accessed on 3 March 2022). We set the number of neighbors to 20 and the cluster resolution to 0.8, and used the ScoreJackStraw() function and 0.05 (the bound of P-value) to determine the number of principal components.
7. SC3 [51] is a single cell cluster tool combining multiple clustering solutions through a consensus approach. SC3 was downloaded from https://github.com/hemberg-lab/SC3 (accessed on 3 March 2022) and ran with default parameters. For example, gene_filter=FALSE, pct_dropout_min=10, pct_dropout_max=9, d_region_min=0.0 and d_region_max=0.07.

In scSPaC and sscSPaC, there are several parameters to be set, such as the top *N* HVGs, the number of reduced dimensions *r* (the components in NMF), the number of clusters *K* and the SPL parameters ζ and λ. In our experiment, we selected the top 1000 highly variable genes by default to conduct clustering analysis. In Section 3.2, we discuss the impact of high variable gene numbers on clustering performance in detail. Considering that HVGs are chosen to reduce the dimensionality of the genes in this study, the effects of the components in NMF on the results are not discussed in this study. The number of real cell classes in the dataset was used uniformly as the component dimension *r* of NMF. In Section 3.3, we discuss the impact of number of clusters on the results of the proposed scSPaC in this work. We use adjusted rand index (ARI), purity and normalized mutual information (NMI) in Section 2.5 to evaluate the clustering results. The results of all experiments are the means and standard deviations calculated from 20 repetitions.

Table 2 shows the clustering results on simulated datasets. For the simulation data, our method achieved the highest purity, indicating that the cells can be well clustered into some higher purity classes. For ARI and NMI, we also achieved the highest performance. SC3 is a very competitive approach, having the best clustering performance among the baseline algorithms.

**Figure 2:**
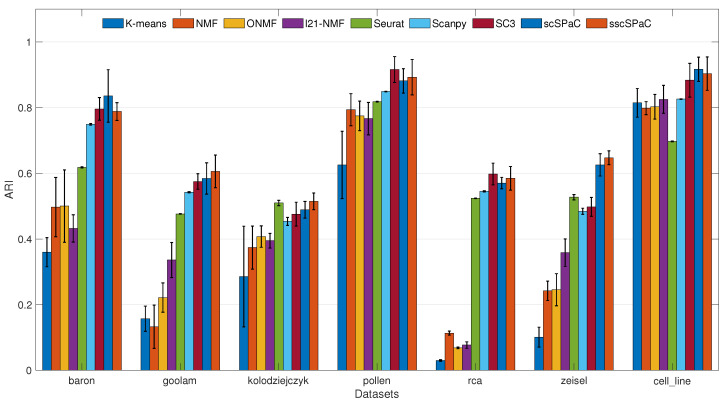
ARI for all test datasets in this study. Bar: average ARI; Errbar: standard deviation of ARI values for 20 runs.

We tested the results of our two methods, scSPaC and sscSPaC against the seven benchmark methods on seven real scRNA-seq datasets. Clustering results for ARI on real scRNA-seq data are shown in Table 3 and Figure 2. On most of the test datasets, we had a 3–4% improvement in ARI. In the zeisel dataset, we had close to 15 point improvements in our evaluation metrics, which shows that our proposed algorithm works well on large-scale datasets. Although SC3 was a very competitive method on both pollen and rca datasets. Our sscSPaC model achieved the second best clustering performance. The results of the other two evaluation indicators purity and NMI are shown in Table 4 and Table 5. It can also be confirmed from the tables that our method achieved the best or second best results in most cases compared with the comparison methods.

### 3.2. Different Numbers of Variable Genes Were Selected for Comparison

To do clustering analysis, we chose the top 1000 highly variable genes by default in our methods. In fact, highly variable genes can collect more biological information than lowly variable genes with little effect on cell type determination [52]. Furthermore, we could lower the model and temporal complexity of our clustering methods by picking highly variable genes. We varied the number of highly variable genes from 200 to 2500 and used scSPaC and sscSPaC on seven real datasets to see how they affected the outcomes.

**Figure 3:**
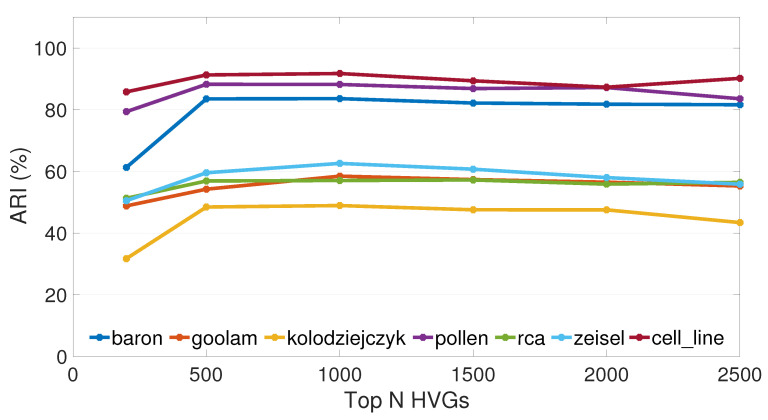
The clustering performance (ARI) with different high variable genes (HVGs). Each broken line represents the ARI of a dataset with 200–2500 high variable genes.

We use the broken line graph in Figure 3 to show the ARI values of seven real datasets by selecting 200, 500, 1000, 1500, 2000 or 2500 highly variable genes. Overall, the performance of 200 high variable genes was somewhat poorer than the other five cases, and the mean values of the other five sets of results did not appear to differ much. In most of the datasets, the results of our scSPaC decreased when more than two thousand HVGs were selected, so only up to a maximum of 2500 HVGs were tested in this study. However, in most datasets, the average ARI computed for 1000 HVGs was still the greatest, so we proposed to use the first 1000 high variable genes for clustering in preference.

### 3.3. Accuracy in Estimating the Number of Clusters

As the number of cell types in a real scRNA-seq dataset is usually unknown, most similarity-based clustering methods require the number of clusters to be specified, and an accurate estimate of the optimal number of cell types is critical to identifying cell types on a real dataset. In this section, we used Scanpy [34], a community detection-based tool that includes an efficient method for partitioning the network into discrete clusters that has been shown to be reliable for forecasting the number of cell types.

In order to evaluate the accuracy of our method in estimating the correct number of populations, the proposed scSPaC in this study searched for the optimal number of clusters around K (from K − 3 to K + 3). K is the number of clusters estimated by Scanpy. As K increases, our model was robust. We recommend that users initialize a slightly larger number of clusters. Table 6 shows the details of how we determined the number of clusters in our model scSPaC. Perhaps it may be more reasonable to add some biological information when analyzing the number of clusters in scRNA-seq data, and combine it with other downstream analysis, such as marker gene identification.

### 3.4. Clustering Pulmonary Alveolar Type II, Clara and Ependymal Cells of Human ScRNA-seq Data

**Figure 4:**
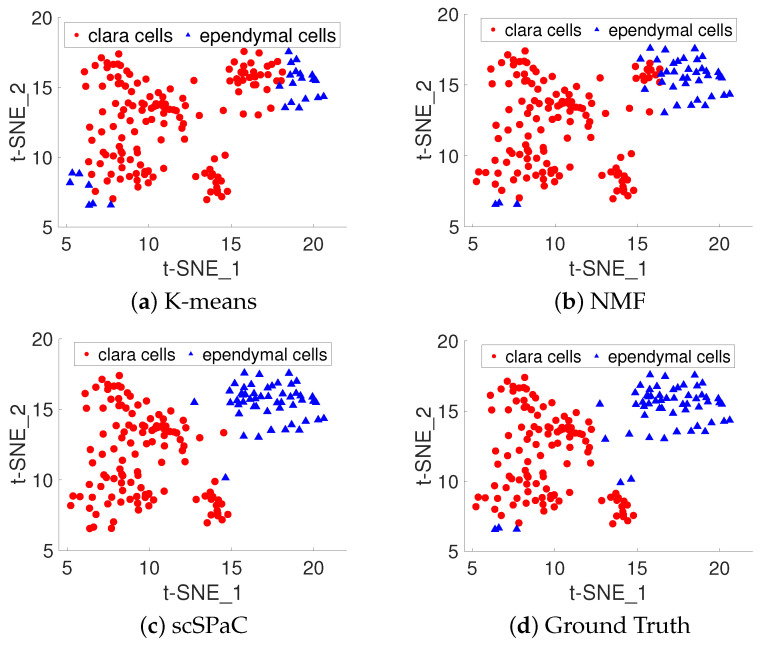
t-SNE for pulmonary alveolar type II, clara and ependymal cells of human scRNA-seq data cluster results. The red filled circles represent clara cells and the blue filled triangles represent ependymal cells. (**a**) t-SNE for K-means; (**b**) t-SNE for origin NMF; (**c**) t-SNE for single cell self-paced clustering (scSPaC); (**d**) t-SNE for ground truth.

To fully examine the validity of scSPaC on different single cell data, we tested the algorithm on human scRNA-seq data. In this section, we focus on the enhancement of the original algorithm in the single cell domain by the addition of self-paced learning. For the sake of simplicity and visualization of the results, we selected human data containing only two cell types. The dataset contains two types of cell lines (113 clara cells and 58 ependymal cells in the human scRNA-seq data) [53]. We use the provided cell type labels as a benchmark for evaluating the performances of the clustering methods. Figure 4 shows the cluster results for t-SNE targeting pulmonary alveolar type II, clara and ependymal cells of human scRNA-seq data. Clara cells are shown in red and ependymal cells in blue. As can be seen in the figure, our scSPaC is more advantageous near boundary lines between clusters. SARS-CoV-2 infection of alveolar epithelial type 2 cells (AT2s) is a defining feature of severe COVID-19 pneumonia [54]. For human lung alveolar type II, our model performs a decent job of discriminating between these clara and ependymal cells, which could help with drug development.

## 4. Conclusions

The advent of single cell sequencing technology provides an opportunity to reveal cellular heterogeneity. In this study, a new sample selection strategy, self-paced learning, is introduced for scRNA-seq data clustering, which solves the clustering problem: that these comparison algorithms are easy to fall into local optimum. Cells are grouped into clustered samples from easy to hard based on the loss of initialization. In order to reduce the impacts of noise and outliers on clustering results, two non-negative matrix factorization algorithms based on self-paced learning were introduced in this work. We test scSPaC and sscSPaC on both simulated and real scRNA-seq data. The state-of-the-art performance was achieved compared to baseline clustering algorithms. In a case study, our scSPaC was more advantageous near the clustering dividing line. Deep learning is computationally expensive compared to traditional machine learning, needing a huge amount of memory and processing resources, and it is difficult to adapt to new situations. It is difficult to put into words and is not totally understood [55,56]. As a result, we only talked about the applicability of the self paced learning technique to scRNA-seq data in the traditional machine learning model in this study.

Although the newly proposed methods scSPaC and sscSPaC performed well in identifying new cell types, it still has some shortcomings. For example, the computational complexity is relatively high, and it requires a relatively long time and large memory size, especially for large-scale datasets. Based on the proposed computational framework, some future improvements will be considered, for example, designing a more elegant regularization term or a deep learning framework to characterize the non-linear relationship among single cells and improve similarity learning by integrating additional multi-omics data.

## Figures and Tables

**Table 1:** A summary of the scRNA-seq datasets used in this study.

Datasets	# Clusters	# Cells	# Genes	Cluster Size	Reference
simulated data	2	200	22002	100+100	Splatter [25]
baron	14	1937	20125	$\begin{array}{c} ccc110+51+236+872+214+120+130+13+70+14+8+92+5+2 \end{array}$	GSE84133 [26]
kolodziejczyk	3	704	32316	295+159+250	E−MTAB−2600 [27]
pollen	11	301	20367	22+17+37+26 +8+16+54+42 +40+15+24	SRP041736 [28]
rca	7	561	20949	74+55+165+96 +51+47+73	GSE81861 [29]
goolam	5	124	26670	6+16+6+64+32	E−MTAB−3321 [30]
zeisel	9	3005	13845	198+948+175+26+290 +98+60+820+390	GSE60361 [31]
cell lines	4	1047	18666	325+203+381+138	GSE126074 [32]

**Table 2:** Evaluation of clustering performance on simulated data. The highest score for each dataset is shown in **bold** and the second best score is underlined. The values in the table represent the (mean ± std).

Datasets	ARI	Purity	NMI
K-means	0.45 ± 0.93	52.45 ± 2.96	0.89 ± 1.07
NMF	9.92 ± 9.72	64.03 ± 7.78	8.20 ± 7.30
ONMF	0.47 ± 1.01	52.50 ± 3.00	1.00 ± 1.27
$l_{2,1}$-NMF	0.64 ± 0.93	53.78 ± 2.74	1.29 ± 1.18
Seurat	0.00 ± 0.00	54.83 ± 0.06	0.10 ± 0.01
Scanpy	0.20 ± 0.00	57.52 ± 0.08	3.67 ± 0.13
SC3	10.79 ± 0.95	63.68 ± 5.72	9.26 ± 1.09
scSPaC	**26.69 ± 15.44**	**74.35 ± 9.11**	**22.02 ± 12.16**
sscSPaC	10.89 ± 10.40	64.70 ± 8.01	10.47 ± 8.67

**Table 3:** Clustering results for ARI on real scRNA-seq data. The highest score for each dataset is shown in **bold** and the second best score is underlined. scSPaC and sscSPaC are based on the F-norm and $l_{2,1}$-norm NMF with a self-paced learning single cell selection strategy.

Datasets	Baron	Goolam	Kolodziejczyk	Pollen	Rca	Zeisel	Cell Line
K-means	35.96 ± 4.44	15.73 ± 3.83	28.56 ± 15.33	62.55 ± 10.25	3.00 ± 0.22	10.12 ± 3.02	81.46 ± 4.36
NMF	49.73 ± 9.03	13.26 ± 6.60	37.38 ± 6.56	79.39 ± 4.88	11.33 ± 0.61	24.21 ± 2.96	79.85 ± 1.98
ONMF	50.03 ± 11.03	22.16 ± 4.48	40.73 ± 3.26	77.50 ± 4.51	6.83 ± 0.23	24.54 ± 4.89	80.29 ± 3.75
$l_{2,1}$-NMF	43.21 ± 4.16	33.61 ± 5.34	39.48 ± 2.23	76.66 ± 4.92	7.70 ± 0.98	35.83 ± 4.17	82.53 ± 4.26
Seurat	61.82 ± 0.18	47.63 ± 0.08	50.97 ± 0.82	81.82 ± 0.12	52.41 ± 0.08	52.73 ± 0.82	69.73 ± 0.12
Scanpy	74.91 ± 0.24	54.25 ± 0.16	45.37 ± 1.22	84.91 ± 0.10	54.5 ± 0.16	48.46 ± 0.92	82.61 ± 0.10
SC3	79.62 ± 3.44	57.52 ± 2.38	47.57 ± 3.64	**91.62** ± **3.93**	**59.8** ± **3.30**	49.78 ± 2.88	88.36 ± 5.14
scSPaC	**83.57** ± **8.00**	58.43 ± 4.78	48.90 ± 2.55	88.16 ± 3.73	57.02 ± 1.75	62.57 ± 3.39	**91.71** ± **3.68**
sscSPaC	78.84 ± 2.70	**60.6** ± **4.97**	**51.48** ± **2.52**	89.27 ± 5.40	58.49 ± 3.59	**64.75** ± **2.09**	90.37 ± 5.09

**Table 4:** Clustering results for purity on real scRNA-seq data. The highest score for each dataset is shown in **bold** and the second best score is underlined.

Datasets	Baron	Goolam	Kolodziejczyk	Pollen	Rca	Zeisel	Cell Line
K-means	71.95 ± 2.18	57.66 ± 2.82	62.66 ± 9.25	77.54 ± 7.79	30.42 ± 0.19	49.57 ± 2.75	86.43 ± 0.12
NMF	82.56 ± 2.85	59.23 ± 2.79	68.27 ± 3.31	90.02 ± 3.18	31.37 ± 0.54	60.69 ± 2.46	81.74 ± 0.1
ONMF	80.92 ± 4.01	59.23 ± 1.95	69.49 ± 1.4	88.34 ± 3.8	31.01 ± 0.4	58.34 ± 2.26	82.18 ± 0.01
$l_{2,1}$-NMF	92.35 ± 1.47	70.85 ± 4.16	69.22 ± 0.94	91.01 ± 1.74	32.07 ± 1.13	66.3 ± 2.64	87.81 ± 0.1
Seurat	86.15 ± 0.26	72.18 ± 0.04	81.36 ± 0.02	86.15 ± 0.17	72.91 ± 0.01	51.99 ± 0.02	79.52 ± 0.04
Scanpy	87.89 ± 0.06	75.63 ± 0.64	76.44 ± 0.1	93.69 ± 0.06	78.59 ± 0.64	50.68 ± 0.1	88.41 ± 0.03
SC3	90.72 ± 2.28	76.59 ± 2.76	78.13 ± 3.51	94.95 ± 2.76	**86.83** ± **1.08**	78.14 ± 3.01	92.75 ± 0.09
scSPaC	**93.26** ± **2.42**	78.39 ± 1.88	79.03 ± 3.48	**96.21** ± **2.05**	83.22 ± 2.07	**89.05** ± **1.91**	**93.94** ± **1.58**
sscSPaC	92.94 ± 1.39	**83.14** ± **3.7**	**81.85** ± **4.16**	95.83 ± 4.08	84.85 ± 1.92	87.81 ± 2.28	93.18 ± 1.45

**Table 5:** Clustering results for NMI on real scRNA-seq data. The highest score for each dataset is shown in **bold** and the second best score is underlined.

Datasets	Baron	Goolam	Kolodziejczyk	Pollen	Rca	Zeisel	Cell Line
K-means	42.77 ± 3.74	20.2 ± 5.43	32.85 ± 16.3	80.57 ± 6.05	1.39 ± 0.19	19.15 ± 3.56	79.47 ± 2.39
NMF	62.11 ± 4.29	17.34 ± 6.42	42.43 ± 5.59	91.09 ± 2.4	2.62 ± 0.72	35.53 ± 2.22	80.81 ± 3.73
ONMF	60.77 ± 4.89	16.07 ± 3.87	44.33 ± 2.65	89.94 ± 2.84	2.15 ± 0.5	33.48 ± 2.86	80.45 ± 2.11
$l_{2,1}$-NMF	64.75 ± 1.93	51.95 ± 4.02	44.15 ± 1.78	**91.61 ± 1.80**	5.98 ± 1.38	38.76 ± 2.34	84.86 ± 2.91
Seurat	61.57 ± 0.23	43.23 ± 0.07	51.54 ± 0.02	86.11 ± 0.07	38.92 ± 0.04	52.03 ± 0.02	63.62 ± 0.07
Scanpy	73.98 ± 0.22	54.9 ± 0.07	49.56 ± 0.03	89.33 ± 0.12	36.02 ± 0.03	44.25 ± 0.03	80.46 ± 0.12
SC3	80.23 ± 2.72	56.59 ± 3.13	52.67 ± 6.64	91.25 ± 3.4	52.63 ± 3.57	50.01 ± 4.28	82.75 ± 3.14
scSPaC	79.82 ± 3.48	**59.02** ± **5.48**	53.69 ± 3.35	89.09 ± 1.86	51.70 ± 0.41	**63.97** ± **2.12**	89.96 ± 4.51
sscSPaC	**81.94** ± **2.63**	58.48 ± 3.78	**56.81** ± **4.24**	91.42 ± 5.13	**54.96** ± **4.14**	63.41 ± 2.68	**87.23** ± **3.37**

**Table 6:** Changes in ARI values calculated according to different cluster number K in simulated data and 7 real scRNA-seq datasets. “Ref. K” means reference K, the number of provided single cell types. “–” means the number of clusters is less than 2. The bold number indicate the best performance (ARI) of each dataset calculated according to different K.

				ARI around Evaluate K by Scanpy (K ±3)						
Datasets	Ref. K	Evaluate K by Scanpy	Best K by scSPaC	K − 3	K − 2	K − 1	K	K + 1	K + 2	K + 3
simulated data	2	2	2	–	–	–	**0.2662**	0.2448	0.2567	0.2489
baron	14	13	11	0.7808	**0.8357**	0.8319	0.8094	0.7862	0.8249	0.7727
goolam	5	5	5	0.4227	0.4518	0.4615	**0.5843**	0.5758	0.5661	0.5732
Kolodziejczyk	3	8	5	**0.4890**	0.4863	0.4875	0.4671	0.4679	0.4628	0.4605
pollen	11	8	10	**0.7098**	0.7172	0.7893	0.8764	0.8753	**0.8816**	0.8612
Rca	7	9	8	0.5475	0.5419	**0.5702**	0.5671	0.5623	0.5453	0.5286
zeisel	9	13	10	**0.6257**	0.6246	0.6241	0.6078	0.5793	0.5641	0.5632
cell line	4	4	4	–	0.5468	0.7025	**0.9171**	0.9043	0.9102	0.8954

## Data Availability

Not applicable.

## Abbreviations

The following abbreviations are used in this manuscript:

NMF	Nonnegative Matrix Factorization
SPL	Self-Paced Learning
t-SNE	t-Distributed Stochastic Neighbor Embedding
HVGs	High Variable Genes

## References

[1] Tsoucas D., Yuan G.C. (2017). Recent progress in single-cell cancer genomics. Curr. Opin. Genet. Dev..

[2] Huang S. (2009). Non-genetic heterogeneity of cells in development: More than just noise. Development.

[3] Yang L., Liu J., Lu Q., Riggs A.D., Wu X. (2017). SAIC: An iterative clustering approach for analysis of single cell RNA-seq data. BMC Genom..

[4] Marco E., Karp R.L., Guo G., Robson P., Hart A.H., Trippa L., Yuan G.C. (2014). Bifurcation analysis of single-cell gene expression data reveals epigenetic landscape. Proc. Natl. Acad. Sci. USA.

[5] Mieth B., Hockley J.R., Görnitz N., Vidovic M.M.C., Müller K.R., Gutteridge A., Ziemek D. (2019). Using transfer learning from prior reference knowledge to improve the clustering of single-cell RNA-Seq data. Sci. Rep..

[6] Lin P., Troup M., Ho J.W. (2017). CIDR: Ultrafast and accurate clustering through imputation for single-cell RNA-seq data. Genome Biol..

[7] Zhu L., Lei J., Klei L., Devlin B., Roeder K. (2019). Semisoft clustering of single-cell data. Proc. Natl. Acad. Sci. USA.

[8] Van der Maaten L., Hinton G. (2008). Visualizing data using t-SNE. J. Mach. Learn. Res..

[9] Von Luxburg U. (2007). A tutorial on spectral clustering. Stat. Comput..

[10] Hu Y., Li B., Chen F., Qu K. (2021). Single-cell data clustering based on sparse optimization and low-rank matrix factorization. G3.

[11] Wang B., Zhu J., Pierson E., Ramazzotti D., Batzoglou S. (2017). Visualization and analysis of single-cell RNA-seq data by kernel-based similarity learning. Nat. Methods.

[12] Park S., Zhao H. (2018). Spectral clustering based on learning similarity matrix. Bioinformatics.

[13] Kumar M.P., Packer B., Koller D. Self-paced learning for latent variable models. Proceedings of the Conference on Advances in Neural Information Processing Systems.

[14] Bengio Y., Louradour J., Collobert R., Weston J. Curriculum learning. Proceedings of the 26th International Conference on Machine Learning.

[15] Kumar M.P., Turki H., Preston D., Koller D. Learning specific-class segmentation from diverse data. Proceedings of the IEEE International Conference on Computer Vision.

[16] Jiang L., Meng D., Zhao Q., Shan S., Hauptmann A.G. Self-Paced Curriculum Learning. Proceedings of the 29th AAAI Conference on Artificial Intelligence.

[17] Tang K., Ramanathan V., Li F.F., Koller D. Shifting Weights: Adapting Object Detectors from Image to Video. Proceedings of the Conference on Advances in Neural Information Processing Systems.

[18] Huang Z., Ren Y., Pu X., He L. Non-Linear Fusion for Self-Paced Multi-View Clustering. Proceedings of the 29th ACM International Conference on Multimedia.

[19] Ren Y., Zhao P., Xu Z., Yao D. Balanced Self-Paced Learning with Feature Corruption. Proceedings of the International Joint Conference on Neural Networks.

[20] Ghasedi K., Wang X., Deng C., Huang H. Balanced self-paced learning for generative adversarial clustering network. Proceedings of the IEEE/CVF Conference on Computer Vision and Pattern Recognition.

[21] Zheng W., Zhu X., Wen G., Zhu Y., Yu H., Gan J. (2020). Unsupervised feature selection by self-paced learning regularization. Pattern Recognit. Lett..

[22] Ren Y., Que X., Yao D., Xu Z. (2019). Self-paced multi-task clustering. Neurocomputing.

[23] Yu H., Wen G., Gan J., Zheng W., Lei C. (2020). Self-paced learning for k-means clustering algorithm. Pattern Recognit. Lett..

[24] Huang Z., Ren Y., Pu X., Pan L., Yao D., Yu G. (2021). Dual self-paced multi-view clustering. Neural Netw..

[25] Zappia L., Phipson B., Oshlack A. (2017). Splatter: Simulation of single-cell RNA sequencing data. Genome Biol..

[26] Baron M., Veres A., Wolock S.L., Faust A.L., Gaujoux R., Vetere A., Ryu J.H., Wagner B.K., Shen-Orr S.S., Klein A.M. (2016). A single-cell transcriptomic map of the human and mouse pancreas reveals inter-and intra-cell population structure. Cell Syst..

[27] Kolodziejczyk A.A., Kim J.K., Tsang J.C., Ilicic T., Henriksson J., Natarajan K.N., Tuck A.C., Gao X., Bühler M., Liu P. (2015). Single cell RNA-sequencing of pluripotent states unlocks modular transcriptional variation. Cell Stem Cell.

[28] Pollen A.A., Nowakowski T.J., Shuga J., Wang X., Leyrat A.A., Lui J.H., Li N., Szpankowski L., Fowler B., Chen P. (2014). Low-coverage single-cell mRNA sequencing reveals cellular heterogeneity and activated signaling pathways in developing cerebral cortex. Nat. Biotechnol..

[29] Li H., Courtois E.T., Sengupta D., Tan Y., Chen K.H., Goh J.J.L., Kong S.L., Chua C., Hon L.K., Tan W.S. (2017). Reference component analysis of single-cell transcriptomes elucidates cellular heterogeneity in human colorectal tumors. Nat. Genet..

[30] Goolam M., Scialdone A., Graham S.J., Macaulay I.C., Jedrusik A., Hupalowska A., Voet T., Marioni J.C., Zernicka-Goetz M. (2016). Heterogeneity in Oct4 and Sox2 targets biases cell fate in 4-cell mouse embryos. Cell.

[31] Zeisel A., Muñoz-Manchado A.B., Codeluppi S., Lönnerberg P., La Manno G., Juréus A., Marques S., Munguba H., He L., Betsholtz C. (2015). Cell types in the mouse cortex and hippocampus revealed by single-cell RNA-seq. Science.

[32] Chen S., Lake B.B., Zhang K. (2019). High-throughput sequencing of the transcriptome and chromatin accessibility in the same cell. Nat. Biotechnol..

[33] Svensson V., Natarajan K.N., Ly L.H., Miragaia R.J., Labalette C., Macaulay I.C., Cvejic A., Teichmann S.A. (2017). Power analysis of single-cell RNA-sequencing experiments. Nat. Methods.

[34] Wolf F.A., Angerer P., Theis F.J. (2018). SCANPY: Large-scale single-cell gene expression data analysis. Genome Biol..

[35] Lee D.D., Seung H.S. Algorithms for non-negative matrix factorization. Proceedings of the Conference on Advances in Neural Information Processing Systems.

[36] Kong D., Ding C., Huang H. Robust nonnegative matrix factorization using l21-norm. Proceedings of the International on Conference on Information and Knowledge Management.

[37] Gao H., Nie F., Cai W., Huang H. Robust Capped Norm Nonnegative Matrix Factorization. Proceedings of the International on Conference on Information and Knowledge Management.

[38] Zhu X., Zhang Z. (2017). Improved self-paced learning framework for nonnegative matrix factorization. Pattern Recognit. Lett..

[39] Huang S., Zhao P., Ren Y., Li T., Xu Z. (2019). Self-paced and soft-weighted nonnegative matrix factorization for data representation. Knowl.-Based Syst..

[40] Jiang L., Meng D., Mitamura T., Hauptmann A.G. Easy samples first: Self-paced reranking for zero-example multimedia search. Proceedings of the 22nd ACM International Conference on Multimedia.

[41] Rand W.M. (1971). Objective criteria for the evaluation of clustering methods. J. Am. Stat. Assoc..

[42] Hubert L., Arabie P. (1985). Comparing partitions. J. Classif..

[43] Schütze H., Manning C.D., Raghavan P. (2008). Introduction to Information Retrieval.

[44] Strehl A., Ghosh J. (2002). Cluster ensembles—A knowledge reuse framework for combining multiple partitions. J. Mach. Learn. Res..

[45] Qi R., Ma A., Ma Q., Zou Q. (2020). Clustering and classification methods for single-cell RNA-sequencing data. Briefings Bioinform..

[46] Tian L., Dong X., Freytag S., Lê Cao K.A., Su S., JalalAbadi A., Amann-Zalcenstein D., Weber T.S., Seidi A., Jabbari J.S. (2019). Benchmarking single cell RNA-sequencing analysis pipelines using mixture control experiments. Nat. Methods.

[47] Li B., Gould J., Yang Y., Sarkizova S., Tabaka M., Ashenberg O., Rosen Y., Slyper M., Kowalczyk M.S., Villani A.C. (2020). Cumulus provides cloud-based data analysis for large-scale single-cell and single-nucleus RNA-seq. Nat. Methods.

[48] MacQueen J. Some Methods for Classification and Analysis of Multivariate Observations. Proceedings of the 5th Berkeley Symposium on Mathematical Statistics and Probability.

[49] Ding C., Li T., Peng W., Park H. Orthogonal nonnegative matrix t-factorizations for clustering. Proceedings of the 12th ACM SIGKDD International Conference on Knowledge Discovery and Data Mining.

[50] Satija R., Farrell J.A., Gennert D., Schier A.F., Regev A. (2015). Spatial reconstruction of single-cell gene expression data. Nat. Biotechnol..

[51] Kiselev V.Y., Kirschner K., Schaub M.T., Andrews T., Yiu A., Chandra T., Natarajan K.N., Reik W., Barahona M., Green A.R. (2017). SC3: Consensus clustering of single-cell RNA-seq data. Nat. Methods.

[52] Yip S.H., Sham P.C., Wang J. (2019). Evaluation of tools for highly variable gene discovery from single-cell RNA-seq data. Briefings Bioinform..

[53] Franzén O., Gan L.M., Björkegren J.L. (2019). PanglaoDB: A web server for exploration of mouse and human single-cell RNA sequencing data. Database.

[54] Huang J., Hume A.J., Abo K.M., Werder R.B., Villacorta-Martin C., Alysandratos K.D., Beermann M.L., Simone-Roach C., Lindstrom-Vautrin J., Olejnik J. (2020). SARS-CoV-2 infection of pluripotent stem cell-derived human lung alveolar type 2 cells elicits a rapid epithelial-intrinsic inflammatory response. Cell Stem Cell.

[55] Zhang M., Zhang F., Lane N.D., Shu Y., Zeng X., Fang B., Yan S., Xu H. (2020). Deep learning in the era of edge computing: Challenges and opportunities. Fog Comput. Theory Pract..

[56] Janiesch C., Zschech P., Heinrich K. (2021). Machine learning and deep learning. Electron. Mark..

